# Common occurrence of antibacterial agents in human intestinal microbiota

**DOI:** 10.3389/fmicb.2015.00441

**Published:** 2015-05-07

**Authors:** Fatima Drissi, Sylvain Buffet, Didier Raoult, Vicky Merhej

**Affiliations:** Unité de Recherche sur les Maladies Infectieuses et Tropicales Emergentes, UM 63, CNRS 7278, IRD 198, Inserm 1095, Faculté de Médecine, Aix Marseille UniversitéMarseille, France

**Keywords:** bacteriocin, antimicrobial peptides, microbiota, gastrointestinal tract, BUR database

## Abstract

Laboratory experiments have revealed many active mechanisms by which bacteria can inhibit the growth of other organisms. Bacteriocins are a diverse group of natural ribosomally synthesized antimicrobial peptides produced by a wide range of bacteria and which seem to play an important role in mediating competition within bacterial communities. In this study, we have identified and established the structural classification of putative bacteriocins encoded by 317 microbial genomes in the human intestine. On the basis of homologies to available bacteriocin sequences, mainly from lactic acid bacteria, we report the widespread occurrence of bacteriocins across the gut microbiota: 175 bacteriocins were found to be encoded in Firmicutes, 79 in Proteobacteria, 34 in Bacteroidetes, and 25 in Actinobacteria. Bacteriocins from gut bacteria displayed wide differences among phyla with regard to class distribution, net positive charge, hydrophobicity and secondary structure, but the α-helix was the most abundant structure. The peptide structures and physiochemical properties of bacteriocins produced by the most abundant bacteria in the gut, the Firmicutes and the Bacteroidetes, seem to ensure low antibiotic activity and participate in permanent intestinal host defense against the proliferation of harmful bacteria. Meanwhile, the potentially harmful bacteria, including the Proteobacteria, displayed highly effective bacteriocins, probably supporting the virulent character of diseases. These findings highlight the eventual role played by bacteriocins in gut microbial competition and their potential place in antibiotic therapy.

## Introduction

Bacteriocins are natural antimicrobial peptides that are ribosomally synthesized by many bacteria and some Archaea (Cotter et al., 2005; Zacharof and Lovitt, 2012; Yang et al., 2014). Bacteriocins have been shown to successfully inhibit pathogenic bacteria such as *Enterobacter*, *Salmonella*, *Klebsiella*, *Escherichia, Listeria*, *Enterococcus*, and *Clostridium* (Hechard and Sahl, 2002; Snelling, 2005; Kirkup, 2006; Gillor et al., 2008). Therefore, bacteriocins have become increasingly of interest as viable alternative antibiotics to bacteriocins (Cotter et al., 2013). When they were discovered (Gratia, 1925), bacteriocins were thought to be secreted by certain bacteria for the purpose of eliminating competition in the medium. Currently, probiotics are used to enhance the ratio of beneficial to undesirable bacteria in human gastrointestinal microbiota (Fooks and Gibson, 2002) probably via the production of bacteriocins. Probiotics have been proposed to ensure *in situ* production of bacteriocins in order to fight against intestinal infections (Dobson et al., 2012; O’Shea et al., 2012; Schuijt et al., 2013). Altogether, bacteriocins may play a crucial role in determining the composition of gut microbiota (Corr et al., 2007; Angelakis et al., 2013; Million et al., 2013). They are presumed to actively participate in gastrointestinal host defense mechanisms by inhibiting one ecosystem, encouraging another, and offering a competitive advantage to bacteria in the intestinal tract.

A number of web-based software programs including the UniProtKB/Swiss-Prot, BAGEL (de Jong et al., 2006; van Heel et al., 2013) and BACTIBASE (Hammami et al., 2007, 2010) allow the detection and characterization of bacteriocins. Some 300 different bacteriocins have been identified and are available in public databases. They can be classified into three main categories on the basis of common characteristics such as molecular weight, heat stability and primary peptide structure. Class I, lanthionine-containing lantibiotics, are heat-stable polycyclic peptides (<5 kDa). Class II, non-lanthionine-containing bacteriocins are small heat-stable bacteriocins (<10 kDa), are subdivided into subclass IIa (pediocin-like bacteriocins), IIb (two-peptide bacteriocins), IIc (circular bacteriocins), and IId (bacteriocins that do not match the other three categories). Class III are large, heat-labile bacteriocins (>30 kDa) including bacteriolysins (murein hydrolases), colicins and linocins (Klaenhammer, 1993; van Belkum and Stiles, 2000; Kemperman et al., 2003; Cotter et al., 2005, 2013). Considering the potential ecological relevance of bacteriocins in microbial communities, the number of recognized bacteriocins seems to be relatively low. Further studies are needed to assess the production of bacteriocin in microbial communities. Since function prediction is essentially based on homology, the large sequence variability and very small length of certain bacteriocins (less than 30 amino acids) make computational identification of bacteriocins very hard (Whisstock and Lesk, 2003). Thus, bacteriocin databases are most likely incomplete and several genes encoding for bacteriocins are probably annotated as hypothetical proteins.

Here, we focused our analysis on the human gut microbiota. We constructed the most exhaustive database for bacteriocins (‘BUR’ standing for Bacteriocins of the URMITE database) by collecting all currently available sequences from the databases and from NCBI. Protein sequences from this database allowed putative bacteriocins from human gut microbiota to be identified using BLASTp methodology.

## Materials and Methods

### Bacteriocin Database

Bacteriocin peptide sequences were collected from the BAGEL (482 sequences) and BACTIBASE (228 sequences) bacteriocin databases (de Jong et al., 2006; Hammami et al., 2007) as well as from the NCBI database (834 bacteriocins). A thesaurus-search of the NCBI Entrez Gene database using bacteriocin, sakacin, microcin, colicin, and plantaricin and other keywords related to bacteriocins, followed by manual examination of the linked Pubmed article information allowed all the sequences of bacteriocins reported in the literature to be retrieved. The multi-FASTA text file containing protein sequences was manually curated to remove all redundant sequences. Similar bacteriocin sequences from different strains were conserved.

### Strain Sequences Collected

From NCBI and our sequencing platform, we retrieved a total of 641 available genomes for organisms from the gastrointestinal tract (307 whole genomes and 334 draft genomes) belonging to 199 different bacterial genera, including *Lactobacillus* (65 strains), *Streptococcus* (32 strains), *Clostridium* (32 strains), and *Bacillus* (30 strains). Among these genomes, 398 were Gram-positive bacteria, and 243 were Gram-negative bacteria.

### Bacteriocins Prediction

A bidirectional protein BLAST (BLASTp) was performed to identify bacteriocins in the gut genomes. Protein sequences encoding for bacteriocins were used as search strings in a BLASTp series [27] against gut genomes. All primary BLAST hits returning with identity over 50% and coverage over 70% were then subjected to BLASTp against the BUR database using same thresholds.

### Web Interface

**FIGURE 1:**
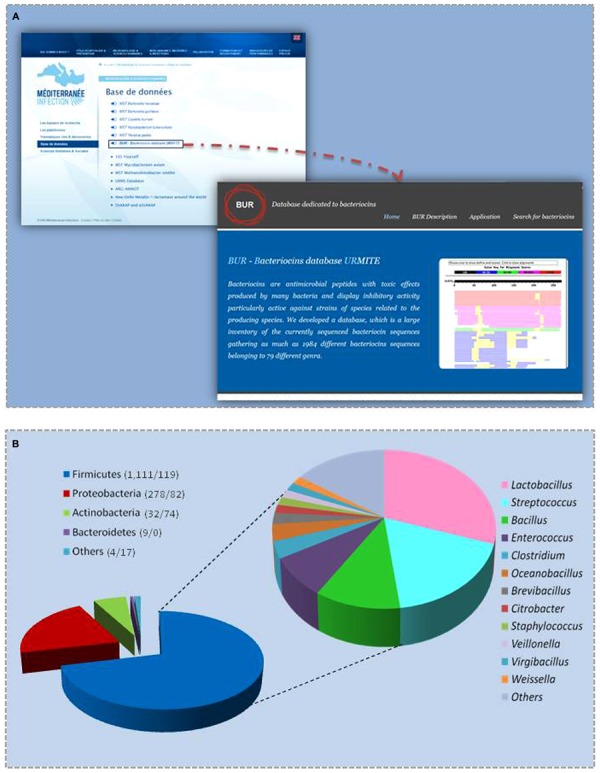
**(A)** Home page access and **(B)** phylum distribution of BUR bacteriocin sequences (numbers in brackets correspond to the number of bacteriocins found in gut bacteria and in other bacteria).

The BUR database runs on a Windows NT platform (Microsoft Windows 2000) with an Apache web server (version 2.0.55). The web server and all parts of the database are hosted at the URMITE in France. The web interface is accessible from the ‘IHU Méditerranée Infection’ page in the database section http://www.mediterranee-infection.com/article.php?larub=143&titre=base-de-donnees (**Figure 1A**). The web interface consists of two main web pages: one homepage with general information about bacteriocins and another page allowing users to search the database using a BLAST algorithm (protein-protein BLAST), version 2.2.13. Sequences that are homologous to the submitted sequences are shown as multiple alignments.

### Information Related to Bacteriocins and Statistical Analysis

General data, such as peptide name, class and microbial data (producer organism, phylum, and Gram staining), were collected for each peptide. Additional physicochemical data including length, mass, amino acid composition, charged residues, hydrophobic (alanine, phenylalanine, glycine, isoleucine, leucine, methionine, proline, valine, and tryptophan) and hydrophilic (cysteine, asparagine, glutamine, serine, threonine, and tyrosine) residues were provided using CLC Genomics v7 software (CLC bio, Aarhus, Denmark). The prediction of bacteriocin potency and spectrum of activity was obtained using Phyre 2 software^1^ (Kelley and Sternberg, 2009). Principal component analysis was performed within the R statistical package^2^ to infer relationships between physiochemical properties and secondary structures within the different bacteriocins classes. SYSTAT 13 software^3^ allowed us to perform statistical analysis using a Chi-squared test. A *p*-value of <0.05 was considered significant.

## Results

The generated database holds 1,359 bacteriocin sequences including 1,050 bacteriocins from Gram-positive bacteria and 309 from Gram-negative bacteria. Of these, 962 are produced by Firmicutes (mainly *Streptococcus* and *Lactobacillus*), 292 by Proteobacteria, 87 by Actinobacteria, eight by Thermotogae, four by Euryarchaeota, three by Aquificae, two by Spirochaetes, and one by Bacteroidetes. The bacteriocin sequences differ greatly in size and composition, with sequences ranging from 10 amino acids (bacteriocins produced by *Lactobacillus curvatus*) to 2,064 amino acids (a bacteriocin produced by *Xylella fastidiosa* strain 9a5c), with average length and weight of 160 amino acids and 18 kDa, respectively. These bacteriocin sequences can be classified into the three classes as follows: 243 in the Class I, 492 in the Class II and 624 in the Class III. Alanine constitutes the most abundant amino acid in the bacteriocin sequences. All these bacteriocins contain an average of 17 glycine residues but no leucine or asparagine residues. Bacteriocin sequences showed a low similarity score of 15% within classes, probably in accordance with the heterogeneity of modes of action and antimicrobial spectra.

When using the available 1,359 sequences of non-redundant protein sequences, we found 367 genes encoding different types of bacteriocins in 317 studied genomes of bacteria from the gastrointestinal tract (Supplementary File 1). Most of these genes were previously annotated as hypothetical proteins in the NCBI database. The 367 bacteriocin sequences identified were added to the BUR database, giving a final database composed of 1,726 peptide sequences (**Figure 1A**). The majority of bacteriocins in the BUR database were produced by Firmicutes (71%), Proteobacteria (21%), Actinobacteria (6%), and Bacteroidetes (0.5%; a total of 1,705 bacteriocins; **Figure 1B**). We observed that 40% of Firmicutes bacteria encode more than three bacteriocins, whereas bacteria from other phyla did not contain more than three bacteriocins. *Lactobacillus, Enterococcus,* and *Streptococcus* spp. encoded one to 24, one to 12 and one to 10 bacteriocins, respectively. *Lactobacillus plantarum* WCFS1, with 24 genes, encoded the largest number of bacteriocins, followed by *L. plantarum* ZJ316 with 23 genes and *L. plantarum* 16 with 21 bacteriocins.

Bacteriocins produced by bacteria found in the gut are very different from those produced by other bacteria with regards to amino acid composition and length. Thus, bacteriocins produced by gut bacteria had significantly smaller percentages of aspartic acid, leucine, arginine, or glutamic acid but higher percentages of lysine and methionine than other bacteriocins (**Table 1**) and they were shorter in length (155 amino acid vs. 298 amino acid, *p* = 0.00). Overall, gut bacteria possessed more class I bacteriocins (44%) than class II (38.6%) or class III (17.3%), whereas other bacteria possessed more class III bacteriocins (59.2%) than class II (27.7%) or class I (13%; *p* = 0.00). Moreover, average hydrophobic residue content and net positive charge of the different classes of bacteriocins from the BUR database were similar (52.5% and +4, respectively).

**Table 1:** Amino acid occurrence in the BUR database.

Amino acid	In gut		Not in gut	
	Number of residues	Percentage of total residues	Number of residues	Percentage of total residues
Alanine^∗^	21,850	9.8	6,055	9.7
Glycine^∗^	20,884	9.3	5,744	9.2
Leucine^∗^	17,214	7.7	5,586	8.9
Serine	16,349	7.3	4,343	6.9
Valine^∗^	15,430	6.9	4,530	7.2
Lysine	15,025	6.7	2,689	4.3
Isoleucine^∗^	13,424	6.0	3,714	5.9
Threonine	12,578	5.6	3,727	6.0
Glutamic Acid	11,830	5.3	3,796	6.1
Aspartic Acid	11,791	5.3	4,291	6.9
Asparagine	11,029	4.9	2,755	4.4
Arginine	8,718	3.9	3,156	5.0
Proline^∗^	8,500	3.8	2,433	3.9
Glutamine	7,957	3.6	2,195	3.5
Tyrosine	7,843	3.5	1,990	3.2
Phenylalanine^∗^	7,615	3.4	1,953	3.1
Methionine^∗^	5,109	2.3	981	1.6
Tryptophan^∗^	4,298	1.9	871	1.4
Histidine	4,069	1.8	1,197	1.9
Cysteine	2,031	0.9	516	0.8

*Hydrophobic amino acids.

**FIGURE 2:**
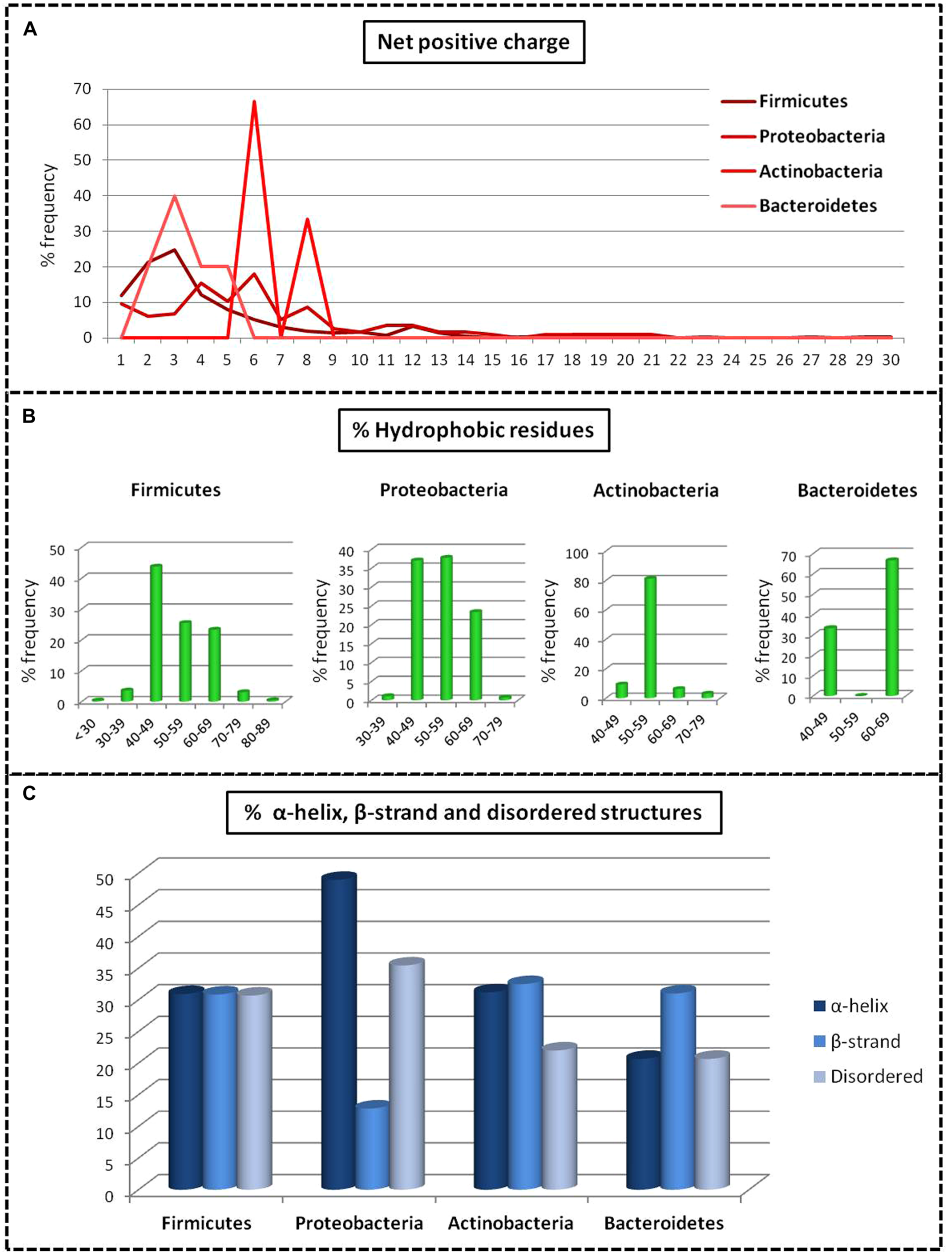
**Comparative analysis of the structures of bacteriocins from gut bacteria produced by the main phyla. (A)** Net positive charge. **(B)** Composition of hydrophobic residues. **(C)** Structural statistics (representation of the mean data).

**FIGURE 3:**
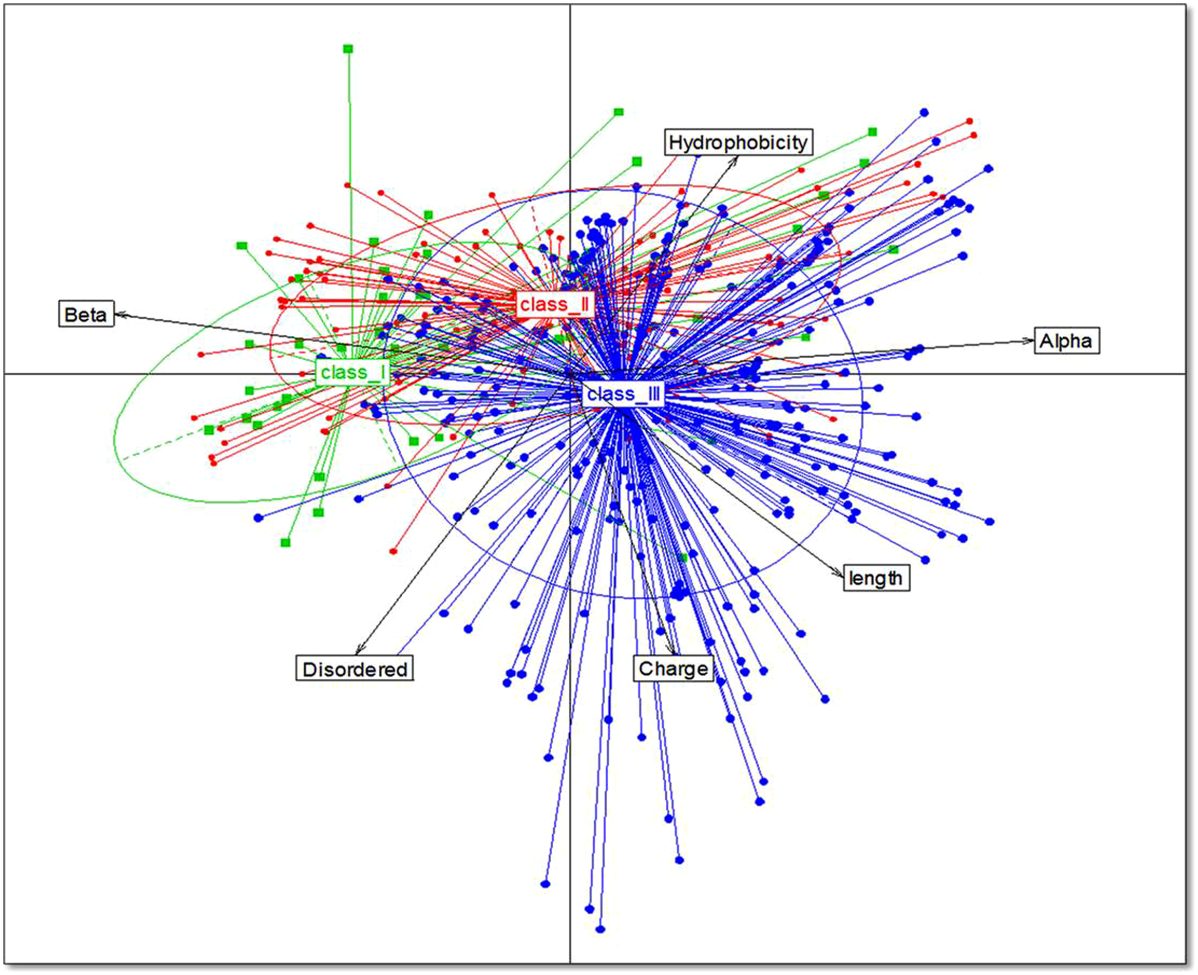
**Relationships between bacteriocins from the different classes**. Data from 1,434 bacteriocins are projected on the first two principal component analysis (PCA) axes.

Analysis of bacteriocins only from gut bacteria revealed significant differences in the structure and physiochemical properties of bacteriocins among the different phyla. Bacteriocins from Firmicutes were distributed fairly evenly across the different classes with 20% in class I, 45% in class II, and 35% in class III. Bacteriocins from Actinobacteria and Proteobacteria belonged primarily to class III (58 and 94%, respectively), and bacteriocins from Bacteroides were exclusively in class III. Proteobacteria had the highest net positive charge (+6), whereas Bacteroidetes had the lowest net positive charge (+3; *p* = 0.01; **Figure 2A**). Although the distribution of the percentage of hydrophobic residues varied widely within the different phyla (**Figure 2B**), bacteriocins from Firmicutes, Proteobacteria, and Actinobacteria all had an average of 53% hydrophobic residues, while Bacteroidetes contained 60% hydrophobic residues. Using homology models, we determined the 2D structure and activity domain for 731 bacteriocins from gut bacteria. Bacteriocins from Firmicutes displayed 31% α-helix, 31% β-strand, and 31% disordered structures, whereas Actinobacteria and Bacteroidetes contained a majority of β-strand structures (32 and 31%, respectively). Bacteriocins from Proteobacteria displayed a significantly higher proportion of α-helix (49%) and disordered structures (35%; *p* = 0.00). Overall, the most abundant peptides were those forming α-helices, with 33% on average (*p* = 0.00; **Figure 2C**). Finally, the principal component analysis revealed that bacteriocin classes were not homogeneous (**Figure 3**). Indeed, even within a class, we observed that bacteriocins displayed different features, except for the length criterion, which was the lowest for class I and the highest for class III.

## Discussion

The present study led to the creation of the largest collection of bacteriocin sequences to date and revealed the abundance of bacteriocins in gastrointestinal tract genomes. These findings help to shed light on the underlying factors that shape the microbial composition of the gut. Indeed, in the highly competitive microbial ecosystem, bacteria seem to foray over each other, producing many signals and substances including bacteriocins (Lahti et al., 2014).

When comparing bacteriocins produced by bacteria found in the gut with other bacteriocins, we showed that they have different amino acid composition (higher levels of lysine and methionine) and also have smaller length, significantly less positive charge, and significantly less hydrophobic residues. However, the α-helical structure was consistent across all bacteriocins. The α-helical peptides are known to be essential for antimicrobial activity (Giangaspero et al., 2001) and have been found to have the broadest spectrum of activity (Dathe and Wieprecht, 1999). Physico-chemical parameters, including size, residue arrangement, charge and hydrophobicity can influence bacteriocin potency and spectrum of activity of α-helical peptides (Giangaspero et al., 2001; Zelezetsky and Tossi, 2006). It has been shown that increasing the charge or hydrophobicity arrangement of a peptide results in potent, broad-range antimicrobial activity (Giangaspero et al., 2001; Zelezetsky and Tossi, 2006).

We found that the Firmicutes and Bacteroidetes, which are some of the most predominant bacterial phyla in the human gut (Eckburg et al., 2005), produce the largest number of bacteriocins. Bacteriocins seem to offer the bacteria within the intestinal tract a competitive advantage (Schuijt et al., 2013). However, these bacteriocins seem to have low antibiotic activity, which may result in moderate control, allowing for a substantial quantity of other bacteria to develop and maintain a fairly balanced diversity within the gut. In contrast, bacteriocins produced by Proteobacteria seem to have higher potential antibacterial activity thanks to cationic charges and α-helices. The relative abundance of disordered structures in Proteobacteria enhances their capacity for interaction depending on the medium and thus ensures potent antibiotic activity in different environments (Zelezetsky and Tossi, 2006). Overall, bacteria from the gut seem to produce a large number of bacteriocins with low activity and small amounts of highly effective bacteriocins. Bacteriocins participate in the establishment of an equilibrium of strength between the predominant commensal bacteria of the microflora and potentially pathogenic opportunistic bacteria.

The majority of gut bacteria were found to produce class I bacteriocins, whereas bacteria not found in the gut were more likely to produce class II bacteriocins. However, we found that bacteriocins displayed different features, even within a class. Considering the relationship between the structure and antimicrobial activity, this finding likely reflects the fact that bacteriocin activity patterns differ considerably within classes. It has been claimed that there is no universal consensus on a method for subdividing the classes of bacteriocins (Ennahar et al., 2000; Reddy et al., 2004). Our findings confirm that classifying bacteriocins into existing classes seems to be uninformative. Indeed, the primary structure of bacteriocins, when there is an appropriate balance between hydrophobicity and net positive charge renders the bacteriocins active toward bacteria. However, selective activity also depends on other parameters; hence, the secondary and tertiary structures of bacteriocins appear to be important for the insertion of peptides through the outer layer of the bacteria and into the phospholipid membrane, due to their contribution to the oligomeric state and volume of the antimicrobial peptides. Overall, the secondary and tertiary criteria may be useful for classifying bacteriocins, for characterizing novel bacteriocins, or for designing novel peptides with high potency against pathogens or with broad antimicrobial spectra.

## Conclusion

Our genome-mining approach allowed the description of the overall production of bacteriocins in the gut microbiota among which Firmicutes that account for a high proportion of the overall population, represent a major group of bacteriocin producers. The physico-chemical characteristics of these bacteriocins predict a low antibiotic activity that may intervene in intestinal host defense against harmful organisms. More investigations of the *in vitro* efficacy, will accurately test for the antibiotic potential and help to elucidate the role played by bacteriocins in the modeling of gut microbial composition.

## Conflict of Interest Statement

The authors declare that the research was conducted in the absence of any commercial or financial relationships that could be construed as a potential conflict of interest.

## Footnotes

1 http://www.sbg.bio.ic.ac.uk/phyre2

2 http://www.r-project.org/

3 http://www.systat.com/

## Abbreviations

BLAST: Basic Local Alignment Search Tool
BUR: bacteriocins of the URMITE
NCBI: National Center for Biotechnology Information
URMITE: Unité de Recherche sur lesMaladies Infectieuses et Tropicales Émergentes
